# Experience of violence among street prostitutes: a qualitative study in Shiraz, Iran

**DOI:** 10.5249/jivr.v11i1.865

**Published:** 2019-01

**Authors:** Ahmad Kalateh Sadati, Nooshin Zarei, Hossein Argasi, Seyed Taghi Heydari

**Affiliations:** ^*a*^Department of Sociology, Yazd University, Yazd, Iran.; ^*b*^Shiraz HIV/AIDS Research Center, Institute of Health, Shiraz University of Medical Sciences, Shiraz, Iran.; ^*c*^Research Consulting Center of Shiraz University of Medical Sciences [RCC], Shiraz University of Medical Sciences, Shiraz, Iran.; ^*d*^Health Policy Research Center, Institute of Health, Shiraz University of Medical Sciences, Shiraz, Iran.

**Keywords:** Sex workers, Violence experience, Qualitative study, Iran

## Abstract

**Background:**

Female Sex workers (FSWs) are a marginalized group. Although some studies have shown various types of violence against sex workers, it is a subject which needs more in-depth knowledge.

**Methods:**

This is a conventional content study conducted on 18 street sex workers in Shiraz, Iran.

**Results:**

The present study observed that sex workers had extensive experience in five forms of violence: physical, barbaric, psychological, sexual, deception and robbery. Moreover, violence was deep-rooted in their previous experiences prior to becoming a prostitute, leading to the formation of yet another type of violence, called hidden slavery with male or female pimps.

**Conclusions:**

To improve the general health of this group, it is recommended that they be supported by social institutions and be provided with psychological consultations.

## Introduction

Violence has several forms, one of which is violence against women. A growing number of studies have documented the high prevalence of intimate partner violence and sexual violence against women worldwide.^1^ Violence against sex workers, street sex workers in particular is a global phenomenon.^2-4^ Sex workers are more likely to experience poor sexual, reproductive and mental health outcomes.^5^ According to Surratt and colleagues, violence against street sex workers is a subculture of violence.^6^ In addition, Sadati et al. showed that murder violence is related to subculture in the context of Shiraz, Iran.^7^ Thus, it seems that such types of violence are related to the fact that women are in a lower position compared to men, which is a culture-related issue. 

There are various sociological theories about violence against women. The critical structuralism theories associated with the structures of the social system are the cause of violence against women. For example, a feminist theory sees intimate partner violence as an expression of gender-based domination of women by men.^8^ Against this view, micro-sociological theories believe that violence is neither a thing, nor a property of individuals or organizations; it is a process that takes place in a great many micro-situations and interactions between concretely situated individuals. ^9^ Between these two approaches, there is the social capital theory about violence. Pierre Bourdieu believes that symbolic power which is related to the structures of the society creates gender domination. The symbolic, in Bourdieu’s view, is a formidable but highly elusive type of power, one that affects a “mysterious alchemy” which symbolically delimits “regions” of social space which leads to “symbolic violence’’.^10^ Symbolic violence is, in some ways, more powerful than physical violence, since it is incorporated even in modes of action and knowledge structures of individuals, imposing the legitimacy spectrum of social order.^11^ However, sometimes the definition of symbolic violence brings it closer to the micro-sociological theory of violence against women. For example, symbolic violence is here defined as the ideas and values of a ruling cultural class (e.g., men), purposefully imposed (often through subconscious means) on a dominated social group such as women.^12^

Surratt and colleagues showed that female sex workers experience is a constant cycle of violence throughout their lives, be it physical, and/or sexual abuse as children, beating, rape, or being threatened with weapons.^6^ Sanders believes that childhood sexual abuse is related to entry into the sex industry at an early age, continual experience of violence, particularly from pimps and clients, as well as street environment risks to which sex workers are exposed.^6^ Another facet of violence is called condom negotiating, which is marginalized in the society, and has no power that can entail the prevalence of HIV/STI infection.^13^ Sanders and Campbell showed that sex workers experience some kinds of violence, namely robbery, non-negotiated sex acts, attempts to remove the condom, offensive language, rudeness and disruptive behavior, and being financially ripped off.^14^

Sex work is a fact in Iran and other parts of the world, where it is related with HIV/AIDS. Recently, the Iranian government has acknowledged FSW as one of the groups most vulnerable to HIV, who urgently need prevention and care services. ^15^ Population increase, economic pressures, and the anomic situation of society in Iran has caused prostitution to become a business.^16^ Limited opportunities, drug dependence and financial needs are among other causes of selling sex.^17^ Although there is no recorded data on its prevalence, the existing estimate for the number of FSW in Iran (80,000 or 0.5% of the adult female population) is based on expert opinion and anecdotal evidence of their presence in certain venues.^18^ Karami et al. believe that the total estimated size of FSWs is 690 (95% CI 633, 747), and about 89.43% of FSWs experience sexual intercourse prior to age 20.^19^ However, the reported 300000 prostitutes, including 45000 in Tehran, has not been confirmed by authorities.^20^

Because this phenomena is illegal in Iran, there is no clear evidence about it. In addition, there is an ambiguous overlap between prostitution and other forms of sexual relationship such as temporary marriage which is legal but informal in the context of Iran,^21^ white marriage^22^ and bondswoman. There is yet another type of illegal sexual relationship, where girls or divorced women who have emotional problems or sexual needs, temporarily go out with a boy and have secret sexual relationships. In such a chaotic system, it is not easy to draw a line between different types of sexual relationship. Despite such complexity and based on the definition of prostitution as a sexual activity for money, it seems that FSWs work in two main locales: street and clubs or flats. Street FSWs are very cheap, while club or flat FSWs are more expensive. However, supply and demand system, age of FSWs, attractiveness, and social problems such as homelessness, poverty, divorce, economical corruptions, and addiction determine the price of selling sex. In such a turbulent market, sex is cheap goods where some FSWs even sell their sex for about 5 dollars or maybe less. Although some studies have been done on the subject, the quality and type of violence against FSWs is yet to be fully investigated. Generally, there are several types of violence, such as self-directed violence, interpersonal violence, and collective violence. Moreover, violence can be of physical, sexual, psychological, deprivation or neglect natures.^23^ Because FSWs are exposed to violence,^24-27^ called intimate partner violence (IPV), therefore it can be situated under the umbrella of gender-based violence and traced to male sexual proprietaries. ^28^ Because there is no evidence about the subject in Iran, this study aimed to explore the experience and topology of violence. 

## Methods 

This is a qualitative content analysis study conducted in 2014, in Shiraz, Iran. Data were collected from 18 street sex workers who referred Drop-in Center (DIC). This center is primarily established for methadone therapy in street addicts, which simultaneously provides services such as educational pamphlets, free consultation and free condoms to prevent the prevalence of HIV/STI. Street sex workers who refer to this center are trained to have safe sex.

Because this center provides real samples, we selected it for the collection of our samples. Women who were informed of the study plan participated in the study for data gathering. After presenting the objectives of the study to sex workers, those ready to participate in the study were interviewed in the private room of the center. They were paid 300,000 Rials for their time, and asked for permission to record their interviews. Interviews started with open-ended questions about their experiences, reasons for entry into sex work and challenges. One of the medical experts was responsible for conducting the interviews, each lasting for 30 min on average. The subjects were interviewed after elucidating the research objectives and setting up an environment of reliability. The interviews were recorded digitally and further transcribed. Based on saturation criteria, 18 subjects were finally selected. 

Data analysis was done based on conventional content analysis. According to Hsieh and Shannon, content analysis included three main approaches which conventional type is done when researcher don’t have any on previous theoretical framework for data analysis.^29^ Thus, this analysis was done according to inductive thermalizing. After extracting the meaning units and exploring the similarities and differences in the data, segments of data were sorted independently into subcategories, and finally the codes were explored. Every theme was, therefore, interpreted according to meaning units, subcategories and codes. Each transcript was coded independently by two researchers. To achieve consensus, they discussed their experiences to ensure that each understood the code and was able to identify segments of the text that best described a theme. Where disagreements were noted, the researchers discussed their difficulties, clarified the difference in their opinions, and agreed to a final classification. Although researchers had previous literature, without any pre-determined theoretical frameworks, all codes were taken from the data through repeated reading. Through this approach, we tried to achieve credibility and authenticity. Member check was used to optimize the validity of qualitative research findings. Due to the sensitivity of the subject, this study was approved by the Ethics Committee of Shiraz University of Medical Sciences.

## Results

About 87 codes, 15 subcategories and 6 categories were extracted from the data. The results of the study showed that all participants experienced at least one type of violence. They experienced so much violence that became natural to them. Specifically, physical and psychological violence were common among them. This experience had started in their childhood or when they got married at an early age and lived with their husbands. Because of their family’s low socioeconomic status and dismantled homes, they were abused for a long time. Being abused as a sex worker is the main source of their fear of society coupled with the fear of customers’ behavior and any event during every sexual encounter. Actually, it can be said that their psyche is traumatized because of the many bad experiences they have been through. The following are the types of violence they had to undergo: physical, barbaric, psychological, sexual, deception and robbery, and hidden slavery. 

**Physical **

Twelve people had experienced physical violence such as being beaten with a stick, belt and a slap on the face. They were violated by their brothers, stepmothers, lovers and husbands. In some cases, these types of experience had begun during childhood. 

"I was too young. When I was a kid, my mom died and my dad got married a year later. My father had several partners and was a drug addict and my stepmother was a bad person. As far as I remember, I was under a lot of pressure and usually beaten" (FSW ID 6).

Experiencing physical violence through brothers can be due to conflicts within the families. 

"After I got divorced, I returned to my father's house. My brother and I used Heroin; Heroin usage is not different in males and females, but he did not like me smoking it, so he always hit me. He would throw me in the shower and take my bra and panties off, soak my body and beat me with his belt buckle" (FSW ID 3).

Many of the participants had experienced physical violence by their husbands or lovers, which was a daily routine, and their customers. 

"Yeah, I have experienced beating. There were 3 or 4 customers who not only did not give me money, but they also beat me" (FSW ID 7).

Generally, experiencing physical violence was common among many of the participants, who expected to encounter physical violence on a daily basis. 

**Barbaric **

Barbaric violence, a type of inhumane physical violence, can become so severe as to threaten life. Generally, this form of violence is common among FSWs, and street FSWs are more bound to be exposed to such kind of violence. Women in such cases experienced branding and violent beating which led to paralysis or near-death conditions. 

"My stepmother wouldn’t let me go to school. One day, she hit me in the head in a way that I fell unconscious; then, I remembered going to bed at night and not being able to stand up the next morning; my hands and feet were paralyzed, a situation I was in for two years."(FSW ID 2).

As this statement shows, the participant had experienced barbaric violence when she was 7 years old. At that time, she was robbed of her childhood in the worst possible way. Barbaric violence was not just limited to their childhood; it further entered their underground world of addiction, drug trafficking and prostitution. The next story is a true story about the barbaric violence of customers against prostitutes. 

"Yeah, customers hit them (prostitutes); they brand their faces with cigarette or pipe in a way that leaves visible marks on their faces. The worst type of violence I was witness to was a woman whose costumer had branded her eye with a burring metal stick. Another girl told me the story of two boys who went with one girl. They had used methamphetamine and were delusional. They thought that the girl was a lamb and they clade her throat. Then, they took her flesh for the person who was guarding the place and told him "we have brought you lamb meat". (FSW ID 4). 

This statement shows three of the worst barbaric behaviors of customers towards FSWs. Since prostitution is an underground work associated with drug and alcohol used by customers, barbaric violence is one of the worst hardships that a prostitute has to endure.

**Psychological **

Psychological violence, also common among prostitutes, can take the forms of permanent fear, feeling of guilt in certain cases, emotional suppression by other family members, and worrying about drug or alcohol abuse on the part of customers which can ultimately lead to emotional or mental trauma. Since SFWs are a marginalized group, they think they do not have the right to defend themselves. So, they have experienced psychological violence throughout their lives. In some cases, psychological violence began from their childhood, even prior to becoming prostitutes. 

"My stepmother used profanity all the time. She was very pessimistic and skeptical. In addition, whenever I wanted to get married she rejected my suitors "(FSW ID 8). 

Living with a dysfunctional family, with its diverse problems, was yet another dimension of psychological violence. Many of the participants had problems such as family conflicts, poverty and addiction which intensified their psychological issues. For example, participant No. 6 used to live in a family of addicts, where all the members, even her brother and his wife were addicts. She was forced to provide drugs for them from a very young age. 

"My sister in-law was an addict. My whole family were addicts, too. My stepfather used to beat me. They always sent me to look for drugs" (FSW ID 6).

Another form of psychological problems is related to prostitution trade. Working as a prostitute without any rights coupled with being weak and fragile impose a great psychological pressure, creating an atmosphere of constant fear. Fear of being caught by the police, the sense of shame if the customer is an acquaintance, and being forced into doing something they do not want to and being beaten if they do not obey are the main psychological conditions that such people have to face. Unprotected sex, and realizing that they have to sleep with more than one person are among other challenges. 

"They deceived us; for example, they said they were only two people, but when we got there, we saw many men waiting for us" (FSW ID 2).

All participants had experienced diverse forms of psychological violence before and after entering prostitution and had lived with continuous fear.

"When I'm having sex with a customer, I feel anxious and concerned that the police might arrest me and take me to jail. I ask my clients to finish quickly. Even when the customer leaves the place, we are scared of the hoodlums" (FSW ID 8).

Fear of contracting sexually transmitted diseases is yet another mental stressor. Most of the customers want to have unprotected sex, so they are constantly worried. According to their statements, most customers request unprotected sex. 

"Some of the customers want to have sex without condom; they say sex with condom is not pleasurable, but I don’t accept" (FSW ID 1).

**Sexual **

Sexual violence is any act or attempt to perform a sexual act through violence or force, also called molestation. Many of the participants used the term 'harassment' which refers to sexual abuse. They try to have sex more than once and request changing the position several times. 

"When we have sex with someone alone, they do whatever they want anal, vaginal, oral, and doggy style, etc…" (FSW ID 4).

Sex workers specifically do not like to have anal and oral sex, although some of them might do it by force. A 36-year-old sex worker said:

"Some of them want to have oral sex; I ask them to use a condom when I suck it for them. Some of them want me to suck until they come. I tell them that this is no porn movie" (FSW ID 10). 

The worst form of sexual violence is when they are faced with a group of men who want to have sex at the same time.

"For example, I had a sexual relationship with one; then three men raped me".

Because of such worries sex workers prefer to have sex with older men. Young customers have many requests, prefer more risky behaviors, and harass the sex workers.

**Deception and robbery**

Act of deception occurs when the prostitute is not paid adequately or at all for her services. Robbery refers to when the sex workers are not only not paid, but also get robbed of their money and possessions after sex. 

"When we go with customers, they usually don’t want to pay and want only to harass us. For example, we say 500,000 Rials, they say wow, why? Am I sleeping with an angel; why would I pay so much money for only ten minutes?" (FSW ID 4).

One of the participants referred to one of her friends who had a bitter experience of sexual abuse with deception and robbery:

"Customers hit her and took her cell phone and money. There were 5 men, each of whom had sex with her and once they were finished, they threw her out." (FSW ID 4).

As this statement shows, prostitutes sometimes experience all forms of violence. 

**Hidden slavery**

As these themes show, prostitutes experience different kinds of violence. Because their trade is illegal, they have no way to defend themselves, which leads to new forms of underground social interaction to get protection against violence. They try to survive with an unusual form of slavery by begging for help from a pimp also called hidden slavery, which refers to sexual or economic advantages that a pimp gets by providing protection for a prostitute. FSW ID 3 is a sex worker who works with her two friends. Fear of potential violence has forced them to work with a male as a pimp.

"We work with a man who acts as our bodyguard. If anyone tries to take advantage, for example request anal sex and sex without condom or tries to harass us, he won't allow them and help us." 

Female pimps play the same role as their male counterpart, except that they do not ask for sexual favor. In the context of this study, female pimps are called “Khale” which means aunt (mother's sister) in English. In Iranian culture, Khale plays a special role in the familyand has an intimate relationship with family members, specifically playing the role of a confident. Family members usually consult Khale regarding psychological, sexual and marital problems. Similarly, in the underground world of prostitution, Khale plays the same role in exchange for money and protection. Based on FSW's statements, they prefer to work with a female pimp, since they are more powerful when it comes to bargaining and protection. Customers pay attention to her words and are afraid of her. Therefore, in the presence of a Khale, customers cannot assault the sex workers and she can force them to use condom.

"It's better to work with a Khale; I feel scared to be alone with a man in the room. Now that I work with a Khale, I feel protected" (FSW ID 11).

However, there is a flip side to this coin; male and female pimps exploit prostitutes so unfairly that hidden slavery is formed. They impose a heavy cost for their service, hence the fact that many prostitutes prefer to work alone or in a group, knowing all the consequences. FSW ID 4 recounted the story of one of her co-workers who preferred to work without a Khale.

"She said her Khale (pimp) is a leech. When I go with a costumer alone, I know that the whole thing is mine. But if I go with a Khale, she takes half. This is not fair" (FSW ID 4).

As this statement shows, underground slavery is unacceptable for sex workers; therefore, they prefer to work alone despite all the potential violence they may have to endure. Underground slavery leads to the violation of their boundaries. Due to this fact, only four participants in this study still work with a pimp. 

## Discussion

The goal of this study was to explore the experience of violence among 18 FSWs. Results showed that their experience was not only limited to the time of prostitution, but many of the participants had experienced several forms of violence and abuse before entering this trade. Nonetheless, due to the illegal nature of this trade, experiencing violence comes full complex. In this context, anyone who has entered this trade is fully prepared and aware of its dangers. They know that in any sexual encounter, at least one potential form of violence may occur, be it physical, sexual, psychological, barbaric, deceptive, or robbery-related. Accordingly, they try to protect themselves by seeking refuge in a male or female pimp, a process called underground slavery. 

On the other hand, since pimps exploit these FSW's unfairly, many of them prefer to take the chance and work alone, being fully aware of its potential hazards. Since they are exposed to violence from different aspects, they live in constant fear, which they have gotten used to. 

Other studies have shown that among sex workers, violence can take different forms, such as emotional, physical and sexual,^2^ financial^30^ and economic , physical and psychological.^31^ In addition, sexual and physical violence among FSW's about negotiation around condom usage and payment are common experienced violences.^32^ Also, Karandikar et al. showed that sex workers reported exploitation from male partners, followed by coercion and ending with intense intimate partner’s violence victimization. ^33^ Our findings indicated other forms of violence, such as barbaric violence and further explored violence leading to hidden slavery. 

The study on 138 Ankara's female sex workers showed that 48.5% were exposed to physical abuse and 13% were exposed to sexual abuse in their childhood.^34^

Also, Okala Bout has studied Kenya's female sex workers and indicated that identifying potentially violent clients, finding safer working areas and minimizing conflict with the police are three skills used to avoid the threats of violence.^32^ Although our study showed that another method of our participants was compliance with underground slavery, they tolerate this despite being exploited. However, the majority of the participants accepted violence because they believed pimps were unfair. 

From a social sciences point of view and in relation to the three theoretical views on violence, it seems that the results of this research are in line with the interactional ^9^ and symbolic violence ^11,12^ theories. Violence among these women is relational because prostitution takes place in underground situations. Thus, the relationship between FSWs and their clients is completely unbalanced, and that is why FSWs have been trying to use Khale or pimp to protect themselves. In addition, results showed that prostitution involves symbolic violence. Prostitution as an unlawful and illegitimate work, does not include any social support and these women do not have any social capital to cling to. Therefore, society represents symbolic violence against these women, which is of course, relevant to the illegitimacy of this behavior.

**Implications **

This study showed that FSWs encounter several forms of violence, of which social support institutions, specifically social service and social emergence teams must be aware. They should be prepared for a timely action for preventing each menacing event, specifically concerning barbaric violence. In addition, these institutions need to geographically know these marginalized people and monitor them as much as possible. Further proposed are providing the material needs of these groups, removing the structural causes of the prostitution, strengthening the family institution and educating them for all threats. Here, the role of social work experts can be conducive to this marginalized group. These vulnerable groups should be monitored and protected against potential hazards as much as possible, as they can become problematic for society in many aspects, such as family values, addiction and sexually transmitted diseases. At the same time, societies have to find a solution based on their cultural values, knowing it is impossible to deny the fact that the trade has been an imperative part of any society. Based on religious evidence and values, it seems that this issue can be rectified also highlighting the pivotal role of clergymen in this scenario.^35^

We recommend that future quantitative research evaluate the frequencies and causes of such forms of violence. In addition, this issue can be considered regarding other sexual relationships in the society, such as couples and other forms of relationships. Because violence in this group (FSW) is related to the general context of the society, other studies are suggested for evaluating the social factors affecting the violence against women in the society.

Among the limitations of the study, there were some of the technical problems during data gathering, and, more importantly, the lack of knowledge about violence among FSW in Iran. Further required are more quantitative studies on the subject, and more information about the experience of violence among all women and men in Iran. 

## Conclusion

In the context of our study, we demonstrated that FSW's are exposed to different forms of violence as a result of being marginalized. These people are continuously exposed to different kinds of violence, such as physical, physiological, sexual, barbaric, and deception, and robbery. As a result of the concomitant fears, they seek refuge in a pimp as a hidden slaver. After a while, when they realize that their pimps are also exploiting them unfairly, they prefer to work independently, fully knowing that they will be exposed to potential violence. In this regard, policy makers can be the determining factors in changing this situation according to religious and cultural values of the society by paying attention to the financial aspect of the problem. 

**Acknowledgment**

This research was supported by a grant No. 92-01-59-5786 from Shiraz HIV/AIDS Research Center, Shiraz University of Medical Sciences, Shiraz, Iran. The researchers would like to thank all of the participants in the study. Also, the authors would like to thank the Research Consulting Center of Shiraz University of Medical Sciences (RCC) for their assistance in editing this article.
